# Antibiotic susceptibility of *Escherichia coli* isolated from neonates admitted to neonatal intensive care units across China from 2015 to 2020

**DOI:** 10.3389/fcimb.2023.1183736

**Published:** 2023-05-22

**Authors:** Ruiqi Xiao, Ying Li, Xiaowei Liu, Yijun Ding, Jidong Lai, Yangfang Li, Wenqing Kang, Peicen Zou, Jie Wang, Yue Du, Jinjing Zhang, Yajuan Wang

**Affiliations:** ^1^ Capital Institute of Pediatrics, Beijing, China; ^2^ Department of Neonatology, Children’s Hospital, Capital Institute of Pediatrics, Beijing, China; ^3^ Beijing Obstetrics and Gynecology Hospital, Capital Medical University, Beijing Maternal and Child Health Care Hospital, Beijing, China; ^4^ Department of Neonatology, Beijing Children’s Hospital, Capital Medical University, National Center for Children’s Health, Beijing, China; ^5^ Department of Neonatology, Women and Children’s Hospital, School of Medicine, Xiamen University, Xiamen, Fujian, China; ^6^ Department of Neonatology, Children’s Hospital of Kunming, Kunming, Yunnan, China; ^7^ Neonatal Intensive Care Unit, Children’s Hospital Affiliated to Zhengzhou University, Henan Children’s Hospital, Zhengzhou, Henan, China

**Keywords:** neonate, neonatal infection, *Escherichia coli*, antimicrobial resistance, MLST, epidemiology

## Abstract

**Background:**

*Escherichia coli* is one of the most common pathogens causing neonatal infections. Recently, the incidence and drug resistance of *E. coli* have increased, posing a major threat to neonatal health. The aim of this study was to describe and analyze the antibiotic resistance and multilocus sequence typing (MLST) characteristics of *E. coli* derived from infants admitted to neonatal intensive care units (NICUs) across China.

**Methods:**

In this study, 370 strains of *E. coli* from neonates were collected. *E. coli* isolated from these specimens were subjected to antimicrobial susceptibility testing (by broth microdilution method) and MLST.

**Results:**

The overall resistance rate was 82.68%, with the highest rate of methicillin/sulfamethoxazole (55.68%) followed by cefotaxime (46.22%). Multiple resistance rate was 36.74%, 132 strains (35.68%) had extended-spectrum β-lactamase (ESBL) phenotype and 5 strains (1.35%) had insensitivity to the tested carbapenem antibiotics. The resistance of *E. coli* isolated from different pathogenicity and different sites of infections varied, strains derived from sputum were significantly more resistant to β-lactams and tetracyclines. Currently, the prevalence spectrum in NICUs was dominated by ST1193, ST95, ST73, ST69 and ST131 across China. And the multidrug resistance of ST410 was the most severe. ST410 had the highest resistance rate to cefotaxime (86.67%), and its most common multidrug resistance pattern was β-lactams + aminoglycosides + quinolones + tetracyclines + sulfonamides.

**Conclusions:**

Substantial proportions of neonatal *E. coli* isolates were severely resistant to commonly administered antibiotics. MLST results can suggest the prevalent characteristics of antibiotic resistance in *E. coli* with different ST types.

## 1 Introduction

Newborns admitted to the Neonatal Intensive Care Units (NICUs), and particularly those born preterm, are at high risk of infection for several reasons, including relative immunocompromise from an immature immune system, prolonged hospitalization, and frequent use of invasive devices and antibiotics (Collins et al., 2018). Infectious diseases are also the main causes of neonatal morbidity and mortality (Zhang et al., 2019; GBD 2019 Diseases and Injuries Collaborators, 2020). Globally, 2.6 million newborns still die each year, with preterm birth and infections the two leading causes. Neonatal sepsis and meningitis were responsible for an estimated 420,000 deaths annually, accounting for 16% of neonatal mortality (Khan et al., 2017).

*Escherichia coli* is one of the most well-adapted and pathogenically versatile bacterial organisms (Riley, 2020). It is the main pathogen causing neonatal meningitis and sepsis especially in developing countries (Tan, 2020; van der Flier, 2021; Wen et al., 2021; Hallmaier-Wacker et al., 2022), also a common pathogen of ventilator associated pneumonia (VAP) in hospitals(Scamardo et al., 2020). According to the data from the Neonatal Monitoring Network released by NICHD (the Eunice Kennedy Shriver National Institute of Child Health and Human Development Neonatal Research Network) in 2020 (Stoll et al., 2020), the most frequent pathogens of early onset neonatal sepsis were *E. coli* (36.6%) and Group B *streptococcus* (30.2%), and *E. coli* mainly occurred in premature infants (51.9%). Besides, a retrospective cohort study in China found that the complications and mortality of *E. coli* meningitis were higher than those of other pathogens (Collaborative Study Group for Neonatal Bacterial Meningitis, 2018).

At the same time, bacterial antimicrobial resistance (AMR) further increases the difficulty of treatment and the speed of transmission of infection—has emerged as one of the leading public health threats of the 21st century (Antimicrobial Resistance Collaborators, 2022). Recent surveillance data from the 2000s indicate that antibiotic resistance to all major antibiotic classes exists among *E. coli* strains. These include the production of extended-spectrum-beta-lactamases (ESBLs) (including TEM, SHV, CMY, and CTX-M types), production of carbapenemases (including KPC, NDM, VIM, OXA-48 and IMP types), resistance to fluoroquinolones, aminoglycosides and trimethoprim-sulfamethoxazole and recently also plasmid-mediated colistin resistance (Paitan, 2018). A multicenter cohort study in the United States showed that the majority of neonatal *E. coli* isolates were insensitive to commonly used antibiotics, with 66.8% of isolates insensitive to ampicillin and 16.8% insensitive to aminoglycosides (Flannery et al., 2021).

One major challenge to tackling AMR is understanding the true burden of resistance and its epidemiological distribution. Neonatal clinicians must balance concerns of inadequate empirical coverage for suspected infection with the risks of indiscriminate antibiotic use. Currently, there is a paucity of contemporary, large-scale, neonatal-specific antibiotic susceptibility data for *E. col*i in China. Therefore, this study retrospectively analyzed the antimicrobial resistance characteristics of 370 clinical isolates of *E. coli* among infants admitted to NICUs across China, aiming to provide reasonable guidance for the use of antibiotics in neonatal infections.

## 2 Methods

### 2.1 Sample origin

Patients hospitalized in the NICUs between November 2015 and October 2020, aged ≤28 days, with *E. coli*-positive cultures from any of the specimens described below were included in this study. Specimens were collected from patients who matched these conditions: blood was collected from sepsis, cerebrospinal fluid was collected from meningitis, sputum was collected from lower respiratory tract infection, gastric fluid was collected from early-onset sepsis, ear secretions, umbilical cord secretions were collected for a routine test from neonates without systemic symptoms. A total of 370 *E. coli* strains isolated from clinical culture-positive specimens across China were ultimately included. The specimens were stored in a refrigerator at -20°C. The study was conducted in accordance with the Declaration of Helsinki, met all ethical requirements, and was approved by the ethics committee of Beijing Children’s Hospital.

### 2.2 Identification of *E. coli* strains and antimicrobial susceptibility testing

MacConkey agar (CM00078) and chromogenic *E. coli* media (EC166) (Beijing Land Bridge Technology Co., Ltd., Beijing, China) were used to isolate and identify *E. coli* strains. The *E. coli* strains were stored in the refrigerator at -80°C. The antimicrobial susceptibility tests was performed by the broth dilution method according to the instructions of the Sensititre TM Gram Negative GNX2F Plate (Thermo Fisher Scientific, USA) and included 21 antimicrobial agents, namely Imipenem, Ertapenem, Doripenem, Meropenem, Aztreonam, Ceftazidime, Cefotaxime, Cefepime, Ticarcillin/clavulanic acid, Piperacillin/tazobactam, Tobramycin, Gentamicin, Amikacin, Levofloxacin, Ciprofloxacin, Doxycycline, Tigecycline, Minocycline, Trimethoprim/sulfamethoxazole, Colistin, and Polymyxin B. Antimicrobial susceptibility testing results were classified as susceptible (S), intermediate (I), or resistant (R), in accordance with Clinical and Laboratory Standards Institute (CLSI), 2023 standards (Clinical and Laboratory Standards Institute. CLSI M100-ED33: 2023 Performance Standards for Antimicrobial Susceptibility Testing, 33rd Edition (2023). M100-ED33). ESBL phenotype, defined as any isolate with at least 1 nonsusceptibility result to cefotaxime, ceftazidime, or cefepime; and carbapenem-resistant Enterobacteriaceae, defined as any isolate with at least 1 nonsusceptibility result to imipenem, meropenem, doripenem, or ertapenem sodium. Definitions of ESBL and carbapenem-resistant Enterobacteriaceae were based on updated Centers for Disease Control and Prevention (CDC) definition (Flannery et al., 2021; Centers for Disease Control and Prevention, Antibiotic/antimicrobial resistance (AR/ARM): biggest threats and data: 2019. AR threats report. https://www.cdc.gov/DrugResistance/Biggest-Threats.html). *E. coli* ATCC 25922 (American Type Culture Collection, Manassas, VA, USA) was used for routine quality control.

### 2.3 DNA extraction

DNA was extracted using bacterial genomic DNA extraction kits (Tiangen Biotech Co., Ltd., Beijing, China). Briefly, after the bacteria were collected by centrifugation, the cell wall was removed by lysozyme digestion. DNA was released from the cell after adding the lysate and proteinase K. Then the binding solution was added to adjust the optimal binding conditions. Next, the solution was transferred to the purification column and centrifuged. DNA was bound to the filter membrane, and impurities such as proteins were filtered out into the filtrate. Residual contaminants and enzyme inhibitors were removed after two washing steps, and the DNA was finally eluted with a small amount of buffer.

### 2.4 Multi-locus sequencing typing

MLST was performed on all isolates. Seven housekeeping genes were targeted as follows: *adk*, *fumC*, *gyrB*, *icd*, *mdh*, *purA*, and *re*cA. The genes were amplified using polymerase chain reaction (PCR) and sent to Tiangen Biotech Co., Ltd., Beijing, China for sequencing. Allelic patterns of these genes were used to determine the sequence type. Sequence data were analyzed based on the *E. coli* MLST database (https://pubmlst.org/organisms/escherichia-spp).

### 2.5 Statistical analysis

Data analysis was performed by SPSS 27.0 (IBM SPSS, Chicago, IL, USA). The χ 2 test was performed for comparing antibiotic and multidrug resistance proportions of *E. coli* strains. Differences with *P* < 0.05 were considered statistically significant.

## 3 Results

### 3.1 General characteristics of *E. coli* strains

Among the 370 *E. coli* strains isolated from newborns aged less than 28 days, 104 were from blood, 31 from cerebrospinal fluid, 96 from sputum, 45 from gastric juice, and 94 from other secretions (ear secretions, umbilical cord secretions). The strains were divided into three groups according to the source, the invasive infection group (n=135) deriving from blood and cerebrospinal fluid, the respiratory tract infection (RTI) group (n=96) consisted of strains from sputum, and the others (n=139) being isolated from gastric juice, ear secretions, and umbilical cord secretions.

### 3.2 Antimicrobial susceptibility testing

In all the *E. coli* isolates, 315 were resistant to at least one antimicrobial drug (the total resistance rate was 82.68%), and the resistance rate of trimethoprim/sulfamethoxazole was the highest (55.68%, 206/370), followed by cefotaxime (46.22%; 171/370), ciprofloxacin (35.41%; 131/370). No *E. coli* strains were found to be resistant to polymyxin B. We defined multidrug resistance in *E. coli* as resistance to at least three distinct antibiotic families and estimated this rate at ∼36.74% (136/370) across all isolates. More details about antimicrobial resistance rates were presented in **Table 1**. And a total of 132 (35.68%) *E. coli* strains had the ESBL phenotype and 5 (1.35%) strains had insensitivity to the carbapenem antibiotics tested (**Table 2**).

**Table 1:** Susceptibility of 370 *E. coli* strains to 21 antimicrobial agents (%).

Antimicrobial agent		R (%)	I (%)	S (%)
**β-lactams**	Imipenem	7 (1.89)	1 (0.27)	362 (97.84)
	Ertapenem	14 (3.78)	3 (0.81)	353 (95.41)
	Doripenem	8 (2.16)	1 (0.27)	361 (97.57)
	Meropenem	9 (2.43)	4 (1.08)	357 (96.49)
	Aztreonam	82 (22.16)	29 (7.84)	259 (70.00)
	Ceftazidime	46 (12.43)	33 (8.92)	291 (78.65)
	Cefotaxime	**171 (46.22)**	2 (0.54)	197 (53.24)
	Cefepime	47 (12.70)	3 (0.81)	320 (86.49)
	Ticarcillin/clavulanic acid	30 (8.11)	92 (24.86)	248 (67.03)
	Piperacillin/tazobactam	16 (4.32)	6 (1.62)	348 (94.05)
**Aminoglycosides**	Tobramycin	35 (9.46)	68 (18.38)	267 (72.16)
	Gentamicin	110 (29.73)	1 (0.27)	259 (70.00)
	Amikacin	3 (0.81)	1 (0.27)	366 (98.92)
**Quinolones**	Levofloxacin	126 (34.05)	152 (41.08)	92 (24.86)
	Ciprofloxacin	**131 (35.41)**	16 (4.32)	223 (60.27)
**Tetracyclines**	Doxycycline	110 (29.73)	95 (25.68)	165 (44.59)
	Tigecycline	1 (0.27)	0 (0.00)	369 (99.73)
	Minocycline	22 (5.95)	38 (10.27)	310 (83.78)
**Sulfonamides**	Trimethoprim/sulfamethoxazole	**206 (55.68)**	0 (0.00)	164 (44.32)
**Polymyxins**	Colistin	2 (0.54)	0 (0.00)	368 (99.46)
	Polymyxin B	0 (0.00)	0 (0.00)	370 (100.00)

The bold values marked representative values with high resistance rates.

**Table 2:** ESBL phenotype and carbapenem resistance status.

	ESBL phenotype^a^	Carbapenems^b^
**No. of strains (%)**	132 (35.68)	5 (1.35)

ESBL, extended-spectrum β-lactamase.

a Includes testing for cefotaxime, ceftazidime, and/or cefepime.

b Includes testing for imipenem, meropenem, doripenem, and/or ertapenem. The bold values marked representative values with high resistance rates.

### 3.3 Resistance of strains from respiratory tract infection, invasive infection and others

According to distinct pathogenic characteristics and various isolation sites, we divided the strains into RTI group, invasive infection group, and others for comparison. *E. coli* isolated from sputum had generally higher resistance to commonly used antibiotics. Almost all those of the β-lactams and tetracyclines were statistically different between the three groups, with aztreonam, ceftazidime, cefotaxime, cefepime, ticarcillin/clavulanic acid, piperacillin/tazobactam, doxycycline, and minocycline were the most significant (*P*<0.001, **Table 3**).

**Table 3:** Comparison of resistance between respiratory tract infection (RTI) group, invasive infection group and others.

Antimicrobial agent		RTI group n=96(%)	Invasive infection group n=135(%)	Others n=139(%)	χ^2^	*P*
**β-lactams**	Imipenem	3(3.1)^a^	4(3.0)^a^	0(0.0)^a^	*	0.083
	Ertapenem	9(9.4)^a^	5(3.7)^a,b^	0(0.0)^b^	13.712	0.001
	Doripenem	3(3.1)^a^	5(3.7)^a^	0(0.0)^a^	*	0.048
	Meropenem	5(5.2)^a^	4(3.0)^a,b^	0(0.0)^b^	*	0.015
	Aztreonam	39(40.6)^a^	23(17.0)^b^	12(8.6)^b^	37.489	**<0.001**
	Ceftazidime	22(22.9)^a^	12(8.9)^b^	8(5.8)^b^	17.900	**<0.001**
	Cefotaxime	70(72.9)^a^	49(36.3)^b^	33(23.7)^b^	58.742	**<0.001**
	Cefepime	28(29.2)^a^	7(5.2)^b^	7(5.0)^b^	40.889	**<0.001**
	Ticarcillin/clavulanic acid	15(15.6)^a^	6(4.4)^b^	5(3.6)^b^	14.744	**<0.001**
	Piperacillin/tazobactam	10(10.4)^a^	4(3.0)^a,b^	1(0.7)^b^	14.378	**<0.001**
**Aminoglycosides**	Tobramycin	11(11.5)^a^	12(8.9)^a^	11(7.9)^a^	0.878	0.645
	Gentamicin	26(27.1)^a^	32(23.7)^a^	48(34.5)^a^	4.084	0.130
	Amikacin	0(0)^a^	0(0)^a^	3(2.2)^a^	*	0.081
**Quinolones**	Levofloxacin	38(39.6)^a^	52(38.5)^a^	32(23.0)^b^	10.005	0.007
	Ciprofloxacin	40(41.7)^a^	51(37.8)^a,b^	36(25.9)^b^	7.387	0.025
**Tetracyclines**	Doxycycline	42(43.8)^a^	23(17.0)^b^	33(23.7)^b^	21.423	**<0.001**
	Tigecycline	0(0)^a^	1(0.7)^a^	0(0.0)^a^	*	0.624
	Minocycline	14(14.6)^a^	7(5.2)^b^	1(0.7)^b^	19.736	**<0.001**
**Sulfonamides**	Trimethoprim/sulfamethoxazole	57(59.4)^a^	77(57.0)^a^	72(51.8)^a^	1.480	0.477
**Polymyxins**	Colistin	1(1.0)^a^	1(0.7)^a^	0(0.0)^a^	*	0.530
	Polymyxin B	0(0)^a^	0(0)^a^	0(0)^a^	*	1.000

* Fisher’s Exact Test. The bold values indicate antibiotics with significant differences among the three groups (p<0.001). Different small letters represent statistically different between groups.

### 3.4 MLST results

Of the 370 *E. coli* neonatal isolates in this study, 313 strains(84.60%) were assigned to 85 known STs, and the remaining 57 strains were unknown ST. Among known STs, the most common sequence type was ST1193 (16.76%; 62/370), followed by ST95 (8.92%; 33/370), ST73 (6.49%; 24/370), ST69 (6.22%; 23/370), ST131 (5.42%; 20/370) and ST410 (4.05%; 15/370). ST410 had the highest multidrug resistance rate (80.00%; 13/16). The multidrug resistance rates of ST410, ST1193 and ST131 were much higher than those of ST95 and ST73 (**Table 4**).

**Table 4:** Multidrug resistance of major E. coli STs.

	ST1193	ST95	ST73	ST69	ST131	ST410
**No. of strains (%)**	**62 (16.76)**	33 (8.92)	24 (6.49)	23 (6.22)	20 (5.42)	15 (4.05)
**No. of multidrug resistant strains (%)**	37 (59.68)	8 (24.24)	6 (25.00)	9 (39.13)	13 (65.00)	**12 (80.00)**

Bolded values indicated the subtype with the highest number proportion (ST1193) and the subtype with the highest multi-drug resistance rate (ST410).

### 3.5 Relationship between antimicrobial resistance and MLST

**Figure 1:**
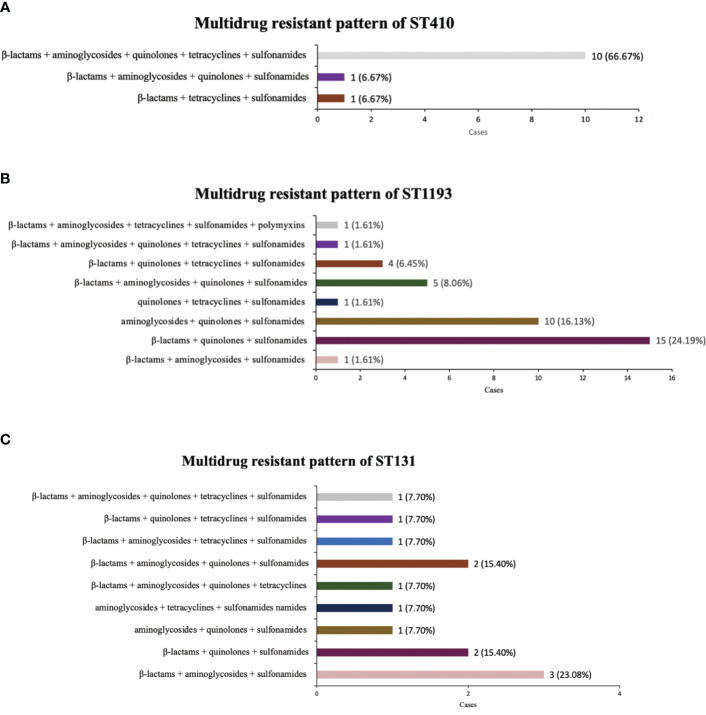
Multi-antibiotics resistance patterns of ST410, ST1193, ST131. **(A)** Composition of antibiotics resistance patterns of ST410. **(B)** Composition of antibiotics resistance patterns of ST1193. **(C)** Composition of antibiotics resistance patterns of ST131.

ST410, ST1193 and ST131 with the top three multidrug resistance rates were all sensitive to amikacin, and were highly sensitive to tigecycline, polymyxin B and colistin. ST410 isolates demonstrated the highest resistance rate to cefotaxime (86.67%; 13/15), and they were found to have serious resistance to β-lactam, quinolones and sulfonamides. The most common multidrug resistance pattern of ST410 was β-lactams + aminoglycosides + quinolones + tetracyclines + sulfonamides (**Figure 1A**). ST1193 exhibited a 90.32% resistance rate to levofloxacin and ciprofloxacin and the common patterns of resistance were β-lactams + quinolones + sulfonamides, aminoglycosides + quinolones + sulfonamides (**Figure 1B**). Among 20 ST131 isolates, 14 (70.00%) were resistant to gentamicin, with 12 resistant to ceftazidime (60.00%), and the most frequent multidrug resistance pattern was β-lactams + aminoglycosides + sulfonamides (**Figure 1C**) (**Table 5**).

**Table 5:** Susceptibility of major *E. coli* STs to 21 antimicrobial agents (%).

Antimicrobial agent		ST1193 (%)	ST131 (%)	ST410 (%)
β-lactams	Imipenem	1 (1.61)	0 (0.00)	3 (20.00)
	Ertapenem	0 (0.00)	0 (0.00)	9 (60.00)
	Doripenem	1 (1.61)	0 (0.00)	4 (26.67)
	Meropenem	0 (0.00)	0 (0.00)	6 (40.00)
	Aztreonam	4 (6.45)	4 (20.00)	10 (66.67)
	Ceftazidime	5 (8.06)	4 (20.00)	11 (73.33)
	Cefotaxime	28 (45.16)	**12 (60.00)**	**13 (86.67)**
	Cefepime	4 (6.45)	3 (15.00)	10 (66.67)
	Ticarcillin/clavulanic acid	2 (3.23)	1 (5.00)	10 (66.67)
	Piperacillin/tazobactam	0 (0.00)	0 (0.00)	12 (80.00)
Aminoglycosides	Tobramycin	6 (9.68)	3 (15.00)	9 (60.00)
	Gentamicin	21 (33.87)	**14 (70.00)**	4 (26.67)
	Amikacin	0 (0.00)	0 (0.00)	0 (0.00)
Quinolones	levofloxacin	**56 (90.32)**	7 (35.00)	11 (73.33)
	Ciprofloxacin	**56 (90.32)**	9 (45.00)	11 (73.33)
Tetracyclines	Doxycycline	11 (17.74)	7 (35.00)	12 (80.00)
	Tigecycline	1 (1.61)	0 (0.00)	0 (0.00)
	Minocycline	1 (1.61)	0 (0.00)	9 (60.00)
Sulfonamides	Trimethoprim/sulfamethoxazole	47 (75.81)	13 (65.00)	13 (86.67)
Polymyxins	Colistin	1 (1.61)	0 (0.00)	0 (0.00)
	Polymyxin B	0 (0.00)	0 (0.00)	0 (0.00)

The bold values marked representative values with high resistance rates.

## 4 Discussion

*E. coli* is a gram-negative bacillus and resident of the normal intestinal microbiota. However, some pathogenic *E. coli* strains are capable of causing human disease, and can be broadly divided into two groups, extraintestinal pathogenic *E. coli* (ExPEC) and intestinal pathogenic *E. coli* (InPEC) (Pokharel et al., 2023). In terms of morbidity and mortality, ExPEC has a great impact on neonatal health, and it has become a common causative agent of neonatal infections, especially invasive infections (Ejiofor et al., 2018). Several pandemics of *E. coli* strains, which are highly virulent and antibiotic resistant, have occurred in recent years (Yair and Gophna, 2018). In addition, selection pressures exerted by antibiotic use, overuse, and misuse are driving a gradual increase in antibiotic resistance and leading to the emergence of multidrug resistant bacterial strains, further accelerating the rate of spread of infection and the difficulty of treatment (Wu et al., 2021).

The emergence of multidrug-resistant *E. coli* has been reported in many countries. And the parallel increase in incidence and frequency of multidrug resistance has raised increasing concerns about the treatment of *E. coli* infections (Dunn et al., 2019). *E. coli* tested in this study generally had a high rate of resistance to commonly used antibiotics, with the highest rate of resistance for methotrexate/sulfamethoxazole (55.68%; 206/370), followed by cefotaxime (46.22%; 171/370), and ciprofloxacin (35.41%; 131/370). The Pediatric Surveillance of Infectious Diseases (ISPED) reported bacterial epidemiology and drug resistance in Chinese children in 2020: *E. coli* resistance to methotrexate/sulfamethoxazole was 54.0%, ceftazidime 49.0%, and ciprofloxacin 41.5%, which is broadly consistent with our findings (Fu et al., 2021). Methotrexate/sulfamethoxazole have been used for decades as effective and inexpensive antimicrobial agents in animals and humans, but extensively resistance has spread widely and rapidly due to the horizontal spread of *sul*1 and *sul*2 genes and expressing dihydropteroate synthases highly resistant to sulfonamide. It is rarely used at present (Sköld, 2001). Notably, the total of 370 neonatal *E. coli* isolates had a multidrug resistance rate of 36.74%, with 35.68% having ESBL phenotype and 5 strains (1.35%) resistant to carbapenem antibiotics. In a cohort study by Flannery et al. of neonatal *E. coli* samples admitted to multicenter NICUs across the United States from 2009 to 2017 (Flannery et al., 2021), cefazolin resistance was 17.1% and ciprofloxacin was 10.2%, with only 5.0% of isolates meeting ESBL phenotype criteria and no resistance to carbapenems was observed. This difference may be due to the different sources of specimens, but it also shows that the form of drug resistance is more severe in China compared to developed countries.

ESBL and CRE have become more prevalent in pediatric patients and are strongly associated with a poorer clinical prognosis, but surveillance data and evidence of medication use for the neonatal population are currently inadequate (Chiotos et al., 2020). Emergence of ESBL-producing Enterobacterales and CRE in neonatal settings is particularly worrisome because such infections may be resistant to most or all conventional antibiotics (Flannery et al., 2022). Horizontal transfer of plasmid-borne ESBL genes causes resistance of *E. coli* to β-lactam antibiotics such as cephalosporin, and cephalosporin treatment failure is a serious problem in infection control worldwide. (Dunn et al., 2019; Ibrahim et al., 2023). A minority of *E. coli* strains were found to be resistant to carbapenem antibiotics such as ertapenem, donipenem, meropenem and imipenem in our study. Carbapenem resistance can result from several mechanisms including, porins coupled with ESBL production, membrane permeability changes *via* mutations in efflux pumps or by hydrolysis of the beta-lactam ring by dedicated carbapenemase enzymes (Paitan, 2018). Treatment options for multidrug-resistant *E. coli* (especially ESBL and CRE) infections in neonates are severely limited. Therefore, there is an urgent need to focus on surveillance, prevention, and management of ESBL and CRE to improve the accuracy of diagnosis of neonatal infections and the rationality of antibiotic use.

We found that *E. coli* with different pathogenicity or isolation sites also differed in drug resistance characteristics. *E. coli* isolated from sputum were generally more resistant to commonly administered antibiotics than those isolated from other sites –even blood and cerebrospinal fluid. Among them, aztreonam, ceftazidime, cefotaxime, cefepime, ticarcillin/clavulanic acid, piperacillin/tazobactam, doxycycline, and minocycline were the most significant(*P* < 0.001). In an adult epidemiological survey conducted in France, pneumonia-specific *E. coli* were also found to be more resistant than commensal isolates to all antimicrobial drugs tested except amikacin and more resistant to cefotaxime and cefoxitin compared to bacteremia isolates (La Combe et al., 2019). *E. coli* is one of the most genetically versatile microorganisms and has the high plasticity of the genome which gives it a tremendous capacity for evolution, resulting in the acquisition of drug resistance genes and virulence factors (Pokharel et al., 2023). Several studies have found that *E. coli* strains containing all virulence genomes had a lower resistance phenotype than that observed in non-virulent *E. coli* strains, and this may be related to the regulation of the bacterial genome (Čurová et al., 2020). As we know, meningitis-associated *E. coli* crosses the blood-brain barrier requiring a combination of virulence factors, such as *OmpA*, *Ibe*, *CNF1* (Yang et al., 2023). We therefore hypothesize that the reason why strains causing invasive infections were instead less resistant than those separated from sputum may be related to the complex regulation between virulence and drug resistance. Moreover, the clinical use of antibiotics has had a selective effect on drug-resistant bacteria (Davies and Davies, 2010). Hospitalized neonates are often in need of respiratory support, which, combined with their immature immune development, makes them highly susceptible to pneumonia. Neonatal pneumonia can be fatal and challenging to diagnose, so empirical antibiotic therapy is often initiated early, further leading to a preferential proliferation of antibiotic-resistant strains in the respiratory tract (Vishnu Bhat and Adhisivam, 2018).

ExPEC strains are comprised of many lineages. MLST is a nucleic acid sequencing-based genotyping method that has been widely used in the study of *E. coli* and the identification of ExPEC-related clonal complexes or lineages. Different ST types have different drug resistance and virulence characteristics (Vanstokstraeten et al., 2022). Manges et al. (2019) using meta-analysis described the type, evolution, distribution and characteristics of ExPEC, which showed that ST131 had the highest proportion, and other major lineages included ST69, ST95, ST10, ST405, ST73, ST410, and ST1193. Among the 370 strains of *E. coli* in the current study, ST1193 (16.76%) was the most prevalent, followed by ST95 (8.92%). In some regions, ST1193 had emerged as a new virulent clone of fluoroquinolone-resistant *E. coli* in several countries (Pitout et al., 2022), and it was also isolated from blood and cerebrospinal fluid specimens of Chinese newborns (Ding et al., 2021). In the bacteremia *E. coli* isolates from newborns in the United States, ST95 and ST131 prevailed; ST1193 emerged recently (Cole et al., 2019), with some similarity to our study, suggesting that there may be an epidemic spectrum in the neonatal population that differs from the adult population.

In this study, we also statistically compared the drug resistance characteristics of isolates from different STs. It is noteworthy that ST410, ST1193 and ST131 had significantly higher multidrug resistance rates than ST95 and ST73, and there was a certain pattern in their resistance profile. ST410 had the most serious multidrug-resistant situation, with very high resistance to β-lactams and quinolones, and its most common multidrug resistance pattern was β-lactams + aminoglycosides + quinolones + tetracyclines + sulfonamides. ST1193 showed the most significant resistance to quinolones (90.32%), in agreement with Johnson et al. (2019), and the common multidrug resistance pattern was β-lactams + quinolones + sulfonamides. ST131 isolate had the highest rate of resistance to gentamicin (70%). Usually, ST131 are reported to produce ESBLs, such as CTX-M-15, and almost all are resistant to fluoroquinolones (Nicolas-Chanoine et al., 2014). The difference may be due to the small number of ST131 *E. coli* isolated in this study, which was not representative enough.

There is an enormous public health burden due to *E. coli* multidrug-resistant high-risk clones such as ST1193, ST131 and ST410. These clones have played pivotal roles in the global spread of AMR. It is notable that ST410, as an emerging multidrug-resistant clone, should raise more serious concerns and be monitored more closely. The results of this study showed the higher level of resistance to fluoroquinolones, cephalosporins, and carbapenems in ST410 compared to other ST types. ST410 belongs to 2 clades namely antimicrobial susceptible ST410-A and ST410-B. Clade B is divided into the following subclades: ST410-B1, ST410-B2 that is associated with fluoroquinolone resistance and *bla*CTX-M-15, while ST410-B3 is linked with fluoroquinolone resistance, *bla*CTX-M-15 and *bla*OXA-181 (Roer et al., 2018). Genomic analysis of ST410 by Chen et al. (2022) revealed that ST410-B2 and ST410-B3, which are resistant to fluoroquinolones, contained identical quinolone resistance determining region (QRDR) mutations, and that the acquisition of these QRDR mutations may be due to a single multiple allele homologous recombination event. And the prevalence of the plasmid-mediated fluoroquinolone resistance determinant *aac(6)-Ib-cr)* was high among ST410 isolates (>90%), especially among the ST410-B3 subclade. Furthermore, the most common ESBLs type in ST410 was CTX-M-15, and the CTX-M-15 genes was mainly carried on the IncF plasmids to move within and between different strains or clones (Pitout et al., 2023). OXA-181 and NDM-5 were the most frequent carbapenemases in ST410 and specifically linked with the ST410-B3 subclade (Roer et al., 2018). The OXA-181 genes were located on near identical broad-host range IncX3 plasmids and NDM-5 genes were located on mosaic narrow-host range IncFII plasmids (i.e. F1:A1:B49) that contained various AMR genes including *bla*CTX-M-15. ST410 high-risk clones acquired MDR determinants (i.e., fluoroquinolone resistance, CTX-M enzyme, carbapenemase) in a stepwise pattern, acting as “hoarders and transmitters” of AMR genes through horizontal and vertical transmission, which together lead to a high level of resistance and risk in ST410. The specimens in our study were obtained from tertiary care hospitals across China, and contained detailed antibiotic susceptibility data for 370 neonatal-specific *E. coli* isolates, which makes the analysis of the prevalence of *E. coli* in Chinese neonates very representative. Nevertheless, this study has some limitations. We only analyzed the antibiotic resistance and susceptibility characteristics of *E. coli* and lacked further classification and analysis of clinical symptoms and diagnostic information. Records on antibiotic dose or frequency of administration and detailed patient-level data, such as maternal antibiotic exposure, gestational age, and mode of delivery, were not available; therefore, we were unable to include these variables in the adjusted analysis.

In conclusion, the results of this study suggest that resistance of *E. coli* clinically isolated from neonates hospitalized in NICUs across China was severe, with a substantial proportion of isolates found to be insensitive to commonly used antibiotics. Particular attention should be paid to the monitoring and management of ESBL-type *E. coli* and strains resistant to carbapenem antibiotics. The resistance phenotype of *E. coli* varied by pathogenicity and by site, with lower respiratory tract infection such as neonatal pneumonia was generally more resistant to antibiotics. Currently, the main prevalent sequence types in the neonatal population in China were ST1193 and ST95, but multidrug resistance was most severe with ST410. Different MLST types existed with different antibiotic resistance patterns, suggesting that the antibiotic resistance characteristics of *E. coli* can be inferred from MLST results.

## Data availability statement

The original contributions presented in the study are included in the article/supplementary material. Further inquiries can be directed to the corresponding author.

## Ethics statement

The studies involving human participants were reviewed and approved by the ethics committee of Beijing Children’s Hospital. Written informed consent to participate in this study was provided by the participants’ legal guardian/next of kin.

## Author contributions

RX drafted the manuscript and did the statistical analysis. YL and XL aggregated and analyzed the data. YJD, JL, YFL and WK completed the data curation, investigation, validation. PZ, JW performed experimental operation and searched for literature research. YD, JZ analyzed the data and edited the manuscript. All authors contributed to the article and approved the submitted version. YW conceptualized and designed the study.
